# Tug of War between Survival and Death: Exploring ATM Function in Cancer

**DOI:** 10.3390/ijms15045388

**Published:** 2014-03-27

**Authors:** Venturina Stagni, Veronica Oropallo, Giulia Fianco, Martina Antonelli, Irene Cinà, Daniela Barilà

**Affiliations:** 1 Laboratory of Cell Signaling, Istituto di Ricovero e Cura a Carattere Scientifico (IRCCS) Fondazione Santa Lucia, 00179 Rome, Italy; E-Mails: venturina.stagni@gmail.com (V.S.); veronica.oropallo@gmail.com (V.O.); giulia.fianco@gmail.com (G.F.); martina.antonelli87@gmail.com (M.A.); irene.cina@libero.it (I.C.); 2 Department of Biology, University of Rome “Tor Vergata”, 00133 Rome, Italy

**Keywords:** ATM kinase, cancer development and therapy, DNA damage response (DDR), apoptosis, receptor tyrosine kinase, oxidative stress, hypoxia and angiogenesis, death receptors, stem cells

## Abstract

Ataxia-telangiectasia mutated (ATM) kinase is a one of the main guardian of genome stability and plays a central role in the DNA damage response (DDR). The deregulation of these pathways is strongly linked to cancer initiation and progression as well as to the development of therapeutic approaches. These observations, along with reports that identify ATM loss of function as an event that may promote tumor initiation and progression, point to ATM as a *bona fide* tumor suppressor. The identification of ATM as a positive modulator of several signalling networks that sustain tumorigenesis, including oxidative stress, hypoxia, receptor tyrosine kinase and AKT serine-threonine kinase activation, raise the question of whether ATM function in cancer may be more complex. This review aims to give a complete overview on the work of several labs that links ATM to the control of the balance between cell survival, proliferation and death in cancer.

## 1. Introduction

Ataxia-telangiectasia mutated (ATM) is a serine threonine kinase originally identified as the product of the gene mutated in ataxia telangiectasia (AT). This gene is located on human chromosome 11q22-23 and codes for a large protein of about 350 kDa characterized by the presence of a phosphatidylinositol 3-kinase (PI3K)-like serine/threonine kinase domain flanked by FAT (FRAP-ATM-TRRAP) and FATC (FAT-*C*-terminal) domains which modulate ATM kinase activity and function. ATM has been identified as a major player of the DNA damage response (DDR) elicited by double strand breaks (DSBs). According to this observation, ATM kinase activity is induced by DSBs and participates in the different responses to this stress including cell cycle arrest, DNA repair apoptosis and senescence, in order to prevent genomic instability. The molecular mechanisms through which ATM exerts its canonical function as a guardian of genomic stability have been largely investigated and have been already reported in several excellent reviews [1–8]. However, novel players are continuously identified and the signaling cascades that allow the choice between cell cycle arrest and DNA repair, or apoptosis or senescence induction, have been only partially elucidated.

Briefly, a model for canonical ATM activation in response to DNA damage has been proposed [9]. According to this model, in response to DNA damage monomeric active ATM in the nucleus is released from an inactive dimeric form. Bakkenist and Kastan proposed that this activation relies on phosphorylation of Ser1981, which would be required to allow the dimer dissociation [9]. Although the occurrence of ATM phosphorylation on Ser1981 is largely used as a marker of activation, *in vitro*, *in vivo* evidence suggest that this event may be dispensable for the induction of ATM activity [10,11]. ATM activation in response to DNA damage relies on the MRN complex (composed by MRE11, RAD50 and NBS1 proteins) which ensures ATM recruitment to the DSBs [12,13]. In response to DNA damage, ATM triggers the activation of a wide range of substrates that allow the modulation of cell cycle arrest, repair, apoptosis or senescence; comprehensive reviews on the molecular mechanisms through which ATM may exert this function have been well covered by several laboratories [1–8] and this theme is therefore not the focus of this work. According to its essential role in the maintenance of genomic stability ATM has been canonically considered a tumor suppressor gene.

## 2. Role of Ataxia-Telangiectasia Mutated (ATM) Deficiency in Mouse Models

Evidence for a role of ATM in tumor initiation and progression comes also from studies aimed at the generation of mouse models in which ATM activity has been genetically modulated. To date several models of *Atm*^−/−^ mice have been generated [14–16]. In all cases *Atm* deficient mice develop thymic lymphoma according to the critical role of ATM in V(D)J recombination, where DSBs physiologically occur and promote a DDR.

More recently, evidence for the ability of ATM kinase dead protein to induce genomic instability *in vivo* has been provided [17,18]. Surprisingly, while ATM deficient mice are born and develop normally, transgenic mice homozygous for a kinase dead version of ATM are embryonically lethal [17,18]. For this reason, the development of conditional knockin mice for ATM kinase dead will be required to further elucidate the role of ATM kinase activity in the development of tumorigenesis *in vivo*. Overall, the ATM knockout and the ATM knockin mouse models provide genetic support *in vivo* for a significant increase in the rate of lymphoid tumor development associated with ATM deficiency.

The central role of ATM in the prevention of genomic instability, as well as the occurrence of the activation of the DDR at early stages of tumor initiation, prompted several groups to investigate the role of ATM expression in several tumor models *in vivo*. ATM loss promotes intestinal tumor development in APC mouse models [19]. Loss of ATM significantly augments Myc-induced tumorigenesis in epithelial tumor (K5-Myc) as well as lymphoma (Eμ-myc) mouse models [20,21]. In this context ATM promotes Myc-induced apoptosis, while its loss of expression results in increased proliferation. Similarly, ATM heterozygosity increases the incidence of breast cancer in TP53 heterozygous mice [22].

## 3. ATM Expression or Activity and Human Cancer

The first clear link between ATM and cancer consists of the observation that AT patients, among other features, display an increased predisposition to the development of lymphoma and leukaemia [23,24]. This feature has been largely explained by the loss of the fundamental role of ATM in the management of DSBs, which physiologically occur in the immune system, and if not repaired lead to the unbalance between proliferation, cell death and differentiation [23,24]. More recently, ATM kinase has been also identified as a modulator of death receptor induced apoptosis, which also plays a crucial role in ensuring the maintenance of the equilibrium and of the functionality of the immune system during development and during the immune response in adult life [25]. ATM deficiency results in the upregulation of the antiapoptotic protein FLIP (FLICE-like inhibitory protein) and confers resistance to programmed cell death elicited by Fas and TRAIL (tumor necrosis factor related apotosis inducing ligand) death receptors, suggesting a novel possible link between ATM loss of expression and the development of leukaemia and lymphomas [26,27].

Several reports suggest that germline ATM mutations enhance cancer predisposition not only when both alleles are affected but also when a single copy is hit. Indeed, heterozygous ATM mutations are associated with up to a fivefold increased risk of breast cancer development, depending on the type of mutation [28,29]. In addition to breast cancer, a next-generation sequencing study recently identified ATM heterozygous mutations in the germline of patients with familial pancreatic cancer and point to ATM as a novel pancreatic ductal adenocarcinoma predisposition gene [30].

Moreover, wide scale studies identified selective changes in ATM expression levels in human tumors. These changes are dependent on different causes among which are ATM somatic gene mutations or deletions. ATM point mutations or deletions are frequently found in chronic lymphocytic leukaemia and are associated with poor prognosis [31].

Interestingly, the analysis of data from the Catalogue of Somatic Mutations in Cancer (COSMIC) identified ATM mutations or deletions in about 5% of about 9000 samples of all types of solid tumors included in this catalogue [32]. In addition to breast cancer [33,34], ATM mutations have been identified significantly in lung [35] and colon cancer [36].

## 4. ATM and the DNA Damage Response (DDR) Activation by Oncogene Stress: Is This Only a Barrier to Cancer Development?

The DNA damage response (DDR) has been suggested to be activated and to act as a barrier at early stages of tumor initiation to prevent tumor progression beyond their early stages [37,38]. This idea is strongly supported by several observations. The first evidence is the identification in human clinical specimens of activated forms of a panel of checkpoint kinases and other proteins that are normally induced in the DDR including ATM, Chk1 and Chk2, histone H2AX (γH2AX) and p53. Importantly, this activation is clearly detectable already at early pre-invasive stages of tumor development. Similarly, DDR activation can be identified in several cell culture and xenograft models as a rather acute response to activated oncogenes, leading to cell cycle arrest, senescence or cell death. DDR markers in this context correlate with the occurrence of markers of senescence and *in vitro* and *in vivo* experiments support the requirement of the DDR for senescence induction in response to replicative stress elicited by oncogenes [39–41]. The mechanisms through which oncogenes may trigger DDR activation have been only partially elucidated. It has been proposed that conditional oncogene expression triggers DNA replication stress, including replication fork collapse and subsequent formation of DSBs and DDR activation. Additional events that occur in cancer, including telomere erosion and induction of reactive oxygen species (ROS) levels, may also trigger the DDR and could therefore play a role to link oncogene overexpression and DDR activation [42].

Several issues still deserve further investigation. For example neither the molecular mechanism that allows some, but not all oncogenes to trigger DDR, nor the significance of DDR activation in a subset of solid tumors, have been clearly elucidated so far. It has been shown that a large number of oncogenes may elicit the DDR, including *cMyc*, *H-ras*, *cyclin E*, *Cdc25A*, *E2F1*, *cdc6*, and *ERBB2* [20,37,38,40,43–45]. Conversely, overexpression of the proto-oncogenic cyclin D1 and loss of the tumor suppressor p16ink4a failed to activate the DDR machinery [46]. Regarding the type of tumors where DDR activation has been detected in human specimens, DDR activation has been identified in major types of human carcinomas, including breast, lung, urinary bladder, colon and prostate tumors, and melanomas, while it is surprisingly absent from testicular germ-cell tumors (TGCTs) [42].

The hypothesis of DDR activation as a cancer barrier, fits well with the observation that DDR activation precedes genetic alterations and genomic instability, which are detected at later stages of cancer progression. In this light, the idea is that an activated DDR would act as a barrier to cancer progression, but at the same time would exert a sort of selective pressure for mutations or epigenetic silencing of checkpoint kinases that may occur at later stages and rescue proliferation of incipient cancer cells, counteract cell death and therefore ultimately promote cancer progression [42]. This hypothesis is in agreement with the tumor suppressor role of many factors that participate in DDR and with their loss of expression or mutation in human cancer.

The functional effect of DDR activation as a barrier to tumor progression deserves further investigation. So far it is mainly based on: (1) correlative evidence: (a) mutations affecting components of the DDR are frequently associated with predisposition to cancer; and (b) co-expression of DDR activation and senescent or apoptosis or cell growth arrest markers; (2) functional requirement of DDR for senescent phenotype induction. Despite these supportive genetic data, causal demonstration that oncogene-induced DDR may suppress tumorigenesis *in vivo* is indeed very limited [47–49]. A role for the DDR as a barrier to tumorigenesis in somatic mammary cells following ErbB2 activation has been proposed using the RCAS/MMTV-TVA mouse model [45]. The authors provide evidence for the induction of an ATM-mediated DDR that activates apoptosis and senescence. However they could not functionally demonstrate a role for ATM in restraining ErbB2-dependent tumorigenesis as ATM-null mice succumb to T cell lymphomas and other diseases rapidly, earlier than mammary tumor development in response to active ErbB2 expression, precluding the *in vivo* functional experiments [45]. More recently, to define the tumor-suppressive function of the DDR in mammary tumor mouse model, Petrini and colleagues utilized, the same somatic ErbB2-induced mammary tumor mouse model, in combination with mutant mouse strains for a panel of genes coding for components of the DDR, such as *Mre11^ATDL/ATDL^*, *Nbs1^DB/DB^*, *Chk2*^−/−^, *Nbs1^DC/DC^* *Chk2*^−/−^, *p53^515C/515C^*, *p53*^−/−^ and *p53BP1*^−/−^, each of which shows defects in checkpoints activation, apoptosis and/or DNA repair [50]. The authors could clearly show that the MRN complex, but not Chk2 and only partially p53, restrains the hyperplastic response and it is required for ErbB2 oncogene-dependent DDR activation and in particular for G2 arrest. Consistently, the *Mre11^ATDL/ATDL^* genetic background promotes NeuT-dependent tumor development and *Mre11^ATDL/ATDL^* mammary tumors display high-grade histopathology and are highly metastatic to lung compared to wild-type (wt) [50].

Recently the identification of an unexpected link between the DDR and the ARF (Alternate reading frame) tumor suppressor suggest a more complicated role of the DDR, and in particular of the ATM kinase, in restraining of tumor progression upon oncogene expression [51]. The authors identify the ATM kinase as a central modulator of the ARF tumor suppressor. Surprisingly, they could show that ATM expression and activity restrains ARF activation. They suggest that ATM may promote PP1 (phosphoprotein phosphatase 1)-dependent dephosphorylation of NPM/B23 allowing the release of ARF which can be targeted for degradation by the ubiquitin ligase ULF (Ubiquitin Ligase for ARF) [52]. Consistently, ARF expression is enhanced at later stages of tumor progression more than activation of the DDR, as shown by the analysis several murine and human tumors, including pancreas, skin, head and neck and urinary bladder cancers [53]. These data suggest the presence of two barriers to tumor progression: (1) the DDR, that would be activated already by a limited replicative stress; and (2) ARF that would require the cross of a higher stress threshold usually supported by multiple oncogene activation. This mechanism would ensure a fine tuning of the stress response, but would also suggest that the expression and the activity of components of the DDR machinery may exert different functions in cancer progression, as well as in cancer response to therapy, depending on the specific oncogenic context. In human clinical samples, loss of ATM expression correlated with higher ARF protein levels and in xenograft experiments inhibition of ATM promotes the tumor-suppressive function of ARF [52].

## 5. ATM Dependent Modulation of Signaling Pathways Outside DDR Implicated in Cancer

During the last 15 years, work of many laboratories identified a role for ATM in the modulation of numerous signalling pathways whose deregulation in cancer may promote tumorigenesis and tumor progression. These include death receptor-induced apoptosis oxidative stress, receptor tyrosine kinases, metabolic changes, hypoxia and angiogenesis, and the reactivation of pathways involved in stem cell maintenance. Support for this comes also from global proteome analysis, which identified about 1000 ATM targets in response to ionizing radiation (IR), among which are several proteins that exert important functions outside of the DNA damage response [54,55]. Importantly, although ATM was originally identified as a nuclear protein, several reports have demonstrated its ability to localize also in the cytoplasm [56–59] and more recently to the mitochondria [60], supporting the idea that ATM may participate in several signaling cascades. Here we will review evidence for a role of ATM in these pathways and discuss their significance in cancer initiation and development.

### 5.1. ATM and Death Receptor Induced Apoptosis

Fas receptor (CD95/APO-1) and TRAIL receptor belong to the death receptor family which plays a crucial role in the transduction of the extrinsic apoptotic signal elicited by specific ligands that upon binding to their target receptors trigger their activation. These signaling pathways play an essential role in the control of the development and homeostasis of the immune system [25], and more in general, participate in the modulation of the equilibrium between proliferation and apoptosis that is often aberrantly deregulated in cancer [61,62]. Fas and TRAIL receptors require the binding of their appropriate ligands (Fas Ligand and TRAIL) to exert downstream death signaling, characterized by the recruitment of several cytosolic proteins to form the death inducing signaling complex (DISC). This event is strictly necessary to catalyze the activation of Caspase-8 and Caspase-10 that in turn initiate and execute the apoptotic cascade. The activation of these initiating caspases is absolutely needed to trigger the apoptotic response to extrinsic death signals and is tightly regulated to avoid undesired cell death. This fine regulation is ensured by several proteins, including the FLIP proteins that compete with procaspases for binding to DISC, preventing their activation and the following apoptotic cascade [63].

Our laboratory identified an interplay between Fas and TRAIL death receptors and the ATM signaling pathway, showing that lymphoblastoid cells derived from AT patients which lack ATM expression are significantly resistant to Fas- and TRAIL- induced apoptosis [26,27]. Loss of endogenous ATM kinase activity results in the aberrant up-regulation of FLIP protein levels. Conversely, the induction of ATM kinase activity by treatments that trigger DNA damage, induce the down-regulation of FLIP protein levels enhancing Fas sensitivity. This finding suggests that ATM loss, reported in several lymphomas, may sustain lymphoma development also because of the consequent resistance to Fas-induced apoptosis. Interestingly, Hodgkin Lymphoma cell lines, characterized by Fas-resistance and by FLIP overexpression, may be sensitized to Fas upon ATM kinase expression, which triggers FLIP down-regulation [26].

Collectively, these data identify a molecular mechanism through which ATM kinase may contribute to the immune system homeostasis and to prevent lymphoma development [26,64].

Recently, we identified a molecular mechanism by which ATM kinase triggers FLIP degradation: ATM enhances ITCH E3-ubiquitin ligase activity, which in turn promotes FLIP ubiquitination and degradation [65]. The observation that ATM is a modulator of FLIP protein levels whose aberrant upregulation has been linked to apoptosis deregulation and to cancer therapy resistance in many tumors [66], provides a novel link between ATM loss of function and the acquired resistance of cancer cells to apoptosis. This issue will be further discussed in the last section of this review.

### 5.2. ATM and Receptor Tyrosine Kinases

Amplification or upregulation of growth factor receptor tyrosine kinases (RTKs) are responsible for human cell transformation, cancer progression and are considered signs of poor prognosis in human cancers [67].

Early work showed a defect in the binding affinity of insulin receptors to insulin in AT cells that expressed low levels of insulin-like growth factor-I receptor (IGF-IR), which could be restored by expression of ATM cDNA [68].

Following these observations, Yang and Kastan reported a cytosolic role for ATM in insulin signaling. They showed that insulin can activate ATM kinase activity and the absence of ATM negatively affected insulin signaling. ATM was shown to phosphorylate 4E-BP1 (eIF-4E-binding protein 1) on S111 in response to insulin stimulation, therefore modulating translation initiation [69]. Furthermore, ATM modulates the translocation of cell surface glucose transporter 4 (GLUT4) that in turn is responsible for glucose uptake in muscle cells [70]. Consistent with this, other studies demonstrated that ATM deficiency causes insulin-resistance, resembles the metabolic syndrome and increase vascular disease [71].

Importantly, ATM has been shown to be a major modulator of AKT activation. AKT plays a key role in pathways related to survival by inhibition of apoptotic signals and promotion of cell cycle progression, with a clear implication in cancer and other pathologies [72]. Moreover, AKT has been shown to be activated in response to a wide variety of growth factors, including insulin or insulin-like growth factor I, EGFR and ERBBs, PDGF and MET receptor [72]. Interestingly, knockout mouse models for *AKT* and *ATM* display similarities in terms of phenotypic abnormalities such as growth retardation, defects in the maturation of the immune system, infertility, resistance to insulin and radiosensitivity [14,73]. Several studies support ATM as a major determinant of full AKT activation, through AKT phosphorylation on serine 473 [70,74–76]. Moreover ATM pharmacological inhibition has been shown to inhibit AKT-dependent prosurvival signal in cancer cells [77,78].

The identification of ATM as a positive regulator of AKT activity strongly supports the idea that ATM may play a role downstream a panel of RTKs and more importantly that in certain contexts ATM may positively modulate cell survival and proliferation and tumor progression.

Recently, evidence for a role of the ATM protein, not only downstream of RTKs, but also upstream as a modulator of RTK expression on the cell surface has been provided [79]. The authors showed that ATM kinase activity sustains the upregulation of MET expression in response to IR, through the activation of the transcription factor NF-κB, which in turn leads to overexpression of MET at the cell surface. This study suggested that ATM activity may help to sustain MET-dependent tumorigenicity [79].

### 5.3. ATM and Oxidative Stress

It is well accepted that oxidative stress may play a role in cancer, as several oncogenes induce oxidative stress, cancer cells generally display abnormalities in metabolism and mitochondrial functionality and are resistant to hypoxia [80].

Over the past two decades, evidence has accumulated that links ATM deficiency to increased oxidative stress. The first hint that ATM may modulate the response to oxidative stress came from the observation that ATM deficiency leads to increased levels of ROS which may play an active role in neurodegeneration linked to AT. Using *Atm*^−/−^ mice, the authors demonstrated that the absence of ATM increased the levels of ROS *in vivo* and caused signs of oxidative stress in the central nervous system, in particular in cells of the cerebellum, the primary site of neurodegeneration in AT patients [81]. More recently, regulation of oxidative stress by ATM has been proposed to play a role in several cellular functions including haematopoietic and neuronal stem cell self-renewal and survival [82–84], RTK activation [85] and hypoxia [86,87], that are often aberrantly regulated in cancer.

The molecular mechanisms that link the induction of ROS in the absence of ATM on one side and the activation of ATM by ROS on the other side have not been fully elucidated.

Recently, a potential role for ATM in the control of an antioxidant response via the pentose phosphate pathway (PPP) has been reported and may explain the increase in oxidative stress shown in ATM null tissues [88]. The authors investigated the link between ATM and PPP and showed that ATM stimulates the PPP by inducing glucose-6-phosphate dehydrogenase (G6PD) activity, which in turn promotes nicotinamide adenine dinucleotide phosphate (NADPH) production and nucleotide synthesis [88].

Since mitochondria are also the major source of intracellular ROS, several laboratories investigated whether ATM may modulate their functionality. Indeed, cells lacking ATM function exhibit alterations in both mitochondrial homeostasis (including defects in mitochondrial structure, decreased membrane potential and respiratory activity) [89,90], and mitochondrial biogenesis pathway mediated by AMPK activity [91,92], that in turn is responsible for elevated ROS production and oxidative stress of AT cells. Recently Valentin-Vega and colleagues reported that also *in vivo*, loss of ATM results in mitochondrial dysfunction in thymocytes, including elevated mitochondrial number and increased mitochondrial ROS production [60].

A milestone in the elucidation of the connection between oxidative stress and ATM has been established by the identification of a direct activation of ATM in the cytoplasm in response to oxidative stress. The authors could demonstrate that in this context, ATM activity is induced via a molecular mechanism entirely different from that which ensures ATM activation by DNA damaging agents [93]. ATM can be directly activated by hydrogen peroxide and forms an active dimer, cross-linked by several disulfide bonds. They identified a critical cysteine residue, C2991, located in the FATC domain of ATM kinase that seems to be essential for activation of ATM by hydrogen peroxide [93].

Although oxidative stress and DNA damage may independently trigger ATM activation and may therefore target different substrates, caution should be taken as oxidative stress and ROS production usually induce DNA damage, and therefore ATM is often exposed to both DNA damage and oxidation simultaneously [85]. This observation supports the idea that the role of ATM in cancer may be very complicated as ATM impinges on several pathways simultaneously that differently modulate tumor initiation and progression.

### 5.4. ATM, Hypoxia and Angiogenesis

Hypoxia is a common phenomenon in cancers. Interestingly, low oxygen tension or hypoxia is a common feature of all solid tumors, and hypoxia is associated with tumor development, malignant progression, metastatic outgrowth, and resistance to therapy and is considered an independent prognostic indicator for poor patient prognosis in various tumor types [94]. A series of complicated mechanisms have been developed at the cellular, tissue as well organismal level, to respond to hypoxia. For example, glycolysis increases to compensate for energy deficiency resulting from compromised oxidative phosphorylation under the hypoxic condition; angiogenesis increases during hypoxia to improve the blood vessel density and subsequently elevate the oxygen supply.

Evidence for a link between ATM and hypoxia has been provided by several laboratories [86,87,95–98]. Importantly, hypoxia has been shown to trigger ATM activation which in turn phosphorylates HIF-1α, a transcription factor that plays a central role in the cellular response to hypoxia, by activating the downstream regulation of metabolism, mitochondrial function and angiogenesis [86]. The authors could show that ATM-dependent phosphorylation of HIF-1α on Ser696 stabilizes the protein under hypoxic conditions, which in turn ensures mTORC1 inhibition and growth suppression, to accommodate the unfavourable hypoxic condition. Interestingly, they suggest that downregulation of ATM levels, which frequently occurs in cancer, may be linked to the escape of the repression of mTORC1 to allow tumor growth in hypoxia. These data are supported by the observation that ATM levels are aberrantly low in the hypoxic regions of pediatric solid tumor xenografts tissues pointing to ATM loss of function as an early step in the genesis of childhood solid tumors [86]. Interestingly mTORC1 negatively regulates autophagy, a catabolic process in which cells deliver cytoplasmic components for degradation to the lysosome. Concomitant with mTORC1 repression by ROS, autophagy increased in cells treated with H_2_O_2_ [99]. Consistently Alexander and colleagues demonstrated that ATM signaling in response to ROS also leads to mTORC1 inhibition and is involved in the consequential induction of autophagy [100]. The connection between autophagy and ATM deserves further investigation but again, it may contribute to enhance the complexity of the ATM role in cancer, as the deregulation of autophagy is considered to contribute to cancer initiation and progression and might represent a novel putative target for cancer therapy [101].

The comprehension of the molecular mechanism through which hypoxia triggers ATM activation deserves further investigation, although it seems independent of the DDR [86]. Interestingly, we have reported that ATM may function as an oxygen sensing protein. The disability of ATM-negative cells to upregulate HIF-1α would be consequential to the impaired sensing of oxygen variations [87].

One of the most important responses to hypoxia is angiogenesis. The expression of HIF-1 correlates with hypoxia-induced angiogenesis as a result of the induction of a major HIF-1 target gene, the vascular endothelial cell growth factor (VEGF), also in tumor development where novel vessels are required to sustain tumor growth [80]. Okuno and colleagues recently reported that *Atm* in mice is activated specifically in immature vessels in response to the accumulation of ROS. Moreover, they could show that *Atm* deficiency lowered tumor angiogenesis and enhanced the antiangiogenic action of agents that block VEGF, suggesting that ATM activation in response to ROS may positively impinge on tumor growth because of its ability to promote angiogenesis [102].

### 5.5. ATM Activity Modulates Stem Cell Survival and Proliferation

It has been largely demonstrated that several pathways that are involved in development and in stem cell proliferation are aberrantly upregulated in cancer. This observation has driven the hypothesis of cancer initiation and propagation by a limited number of cancer cells, termed cancer stem cells, or more correctly, cancer initiating cells [80]. Several studies have been aimed to uncover whether ATM activity may play a role in stem cell maintenance and proliferation. Two main issues will be discussed here: (1) Evidence that support a role for ATM in stem cell survival; (2) reports that suggest a possible role of the DDR in general and specifically of ATM in pathways classically linked to stem cell maintenance.

Regarding the first point, early evidence for a role of ATM in regulation of stem cell survival was first described in neuronal stem cells (NSCs) [103]. In particular ATM expression is abundant in NSCs, but it is gradually reduced as the cells differentiate, suggesting that ATM may play an essential role in NSC survival and function [103]. Recently two groups suggest molecular mechanisms involved in ATM dependent regulation of NSC survival. ATM is required to maintain normal self-renewal and proliferation of NSCs, due to its role in controlling the redox status. Loss of ATM renders NSCs defective for proliferation through oxidative stress-dependent p38 MAPK signalling, suggesting that p38 is a central player in the defective proliferation of *Atm*^−/−^ NSCs induced by oxidative stress [84,104]. Moreover, it has been shown that ATM plays a central role in terminal differentiation of a human neural stem cell line model through its function in DDR [105].

Interestingly, ATM function in the oxidative stress response is also important for homeostasis of normal haematopoietic stem cells (HSCs) [82]. Normal haematopoietic stem cells maintain ROS at lower levels than their mature progeny to prevent cellular differentiation and sustain long-term self-renewal, and the ATM kinase is crucial this process [82]. Recently the Gross Lab suggested that the molecular mechanism involved in this regulation requires the interplay between ATM and the BID (BH3 interacting-domain death agonist) protein, which would link apoptosis, DNA repair and stem-cell quiescence by regulating oxidative stress to enable long-term regenerative capacity [106].

In conclusion these papers suggest that the ability of ATM in the control of oxidative stress may contribute to the regulation of stem cell survival.

Evidence for a role of the ATM protein in signalling pathways classically involved in stem cell maintenance has been provided. Recently we showed that ATM is a novel positive modulator of ITCH E3-ubiquitin ligase activity. A single residue on ITCH protein, S161, which is part of an ATM S/T-Q consensus motif, is required for ATM-dependent activation of ITCH. In fact, we provide *in vitro* and *in vivo* genetic evidence that ATM kinase enhances ITCH enzymatic activity and triggers ubiquitination/degradation of ITCH (itchy E3 ubiquitin protein ligase) substrates such as FLIP-L and JUN [65]. ITCH is a member of the NEDD4-like family of HECT-E3-ubiquitin ligases, a family of proteins that participate in several physiological signaling pathways, including the DNA damage response, tumor necrosis factor (TNFα), Notch and Sonic hedgehog signaling [107]. We could hypothesize that ATM is involved in regulation of other ITCH substrates, among which is the transcription factor GLI-1, a major mediator of the Sonic-Hedgehog (SHH) signalling, which regulates tissue patterning and cell proliferation to ensure the correct execution of developmental pathways and homeostasis of adult tissues [108]. Interestingly, several lines of evidence suggest a putative crosstalk between ATM and the SHH pathway. The first hint comes from the identification of GLI-1 as a substrate of ITCH [109,110] and from the observation that ATM modulates ITCH [65]. A second hint comes from the observation that WIP1, a Ser/Thr phosphatase aberrantly upregulated in cancer that dephosphorylates and modulates, among other targets, also ATM activity [111], is involved in the modulation of the SHH signaling [112]. It has been proposed that during tumorigenesis WIP1 (wild-type p53-induced phosphatase 1) overexpression might contribute to increase proliferative and self-renewing activities of GLI-1, therefore enabling an expansion of cancer stem cells and derived progenitors that sustain tumor growth [112]. Finally, a bidirectional connection between the DDR and GLI-1 has been suggested. Any inappropriate elevation of GLI-1 would induce the DNA damage response, which in turn may decrease GLI-1 activity, ensuring the control of precursors and stem cell numbers and preventing tumorigenesis [113,114]. Future experiments will clarify whether, according to these reports, ATM kinase may directly modulate SHH signaling therefore contributing to the maintenance of stem cell identity.

## 6. Modulation of ATM Activity in Cancer Therapy

Ionizing radiation (IR) and many classical chemotherapeutic drugs trigger ATM kinase activation and their outcome is strongly dependent on the functionality of the DDR in general as well as on ATM expression and activity. The activation of DDR may promote genomic instability in those tumor contexts where its tumor-suppressive function has already been lost, for example following p53 mutation or loss of expression. Importantly, cells derived from AT patients clearly display a significantly augmented sensitivity to IR, pointing to ATM targeting as a valuable tool to modulate the sensitivity of tumors to IR or other chemotherapeutic agents [7,32]. The inhibition of ATM function has therefore been proposed, similarly to the inhibition of other DDR components, as a valuable mechanism to overcome cancer cell resistance to IR or to chemotherapy [32]. So far, four ATM kinase inhibitors have been produced: (1) KU-55933 [115]; (2) CP466722 [116]; (3) KU-60019 [77]; and (4) KU-59403 [117]. Evidence for their ability to sensitise cancer cells to IR has been provided. Moreover, KU-59403 has been shown to be able to inhibit *in vivo* xenograft tumor growth in mice by increasing their responsiveness to chemotherapeutic treatment with etoposide and irinotecan treatment [117], while KU-60019 may radiosensitize glioma in mouse xenograft experiments [118].

Although various ATM inhibitors have been effective in some situations, evidence for off-target effects caused by the currently available inhibitors has also been provided. As an example, KU-55933 has been identified in a screening for compounds that may efficiently inhibit autophagy by targeting Vps34 independently of ATM kinase activity [119].

One major problem behind the idea of targeting ATM activity for cancer therapy comes from the observation that the output of the inhibition of ATM activity does not fully mimic the effect of ATM loss of expression. It has been show that when ATM kinase activity is inhibited, but not when ATM expression is lost, Sister Chromatin Exchange (SCE) and subsequently HR and DSB repair, are impaired [120,121]. Consistently, mouse models genetically modified to express physiological levels of catalytically inactive (kinase dead) ATM protein, display higher levels of genomic instability than that observed in *Atm*^−/−^ models. In addition, mice expressing the kinase dead allele are embryonically lethal, in contrast to the viable KO mice. While further experiments will clarify this issue, it has been suggested that during development inactive *Atm* may impair the function of the DDR to a higher degree than *Atm* deficiency, particularly affecting DNA repair and resulting in higher levels of chromosome instability [122].

Overall, these findings provide support, but at the same time raise some concerns about the pharmacological targeting of ATM in cancer therapy. The support is linked to the possible utility of ATM kinase inhibitors in the treatment of cancer cells upon establishment of the genetic context. For example it has been well documented that, while ATM inhibition sensitizes p53 deficient/mutated tumor cells to chemotherapy, it induces chemoresistance in p53 wt backgrounds [123]. The concern comes from the idea that the enzymatic inactivation of ATM in normal cells, which for example express p53 wt, may substantially enhance genomic instability and therefore generate a boomerang effect that ultimately would promote the development of new tumor growth.

Another possible exploitation of ATM inhibition in cancer therapy is linked to the idea that ATM kinase inhibition may be synthetically lethal with the inhibition of other components of the DDR. For example, since ATM is involved in the homologous recombination, its inhibition may be convenient to sensitize cells to PARP-inhibitors [124,125].

Interestingly, a role for the DDR and ATM activation in the regulation of cancer stem cell survival has been proposed. Cancer stem cells are a small fraction of cells in the tumor, with the abilities of self-renewal and multipotent differentiation that seems to have crucial roles in tumor initiation, metastasis and resistance to anti-cancer therapies [126]. In particular, slow proliferation and elongated cell cycle may allow cancer stem cells to increase time to repair therapy-induced DNA damage before replisome arrival, triggering resistance. Inhibiting the DNA damage checkpoint response may thus release the cell cycle brake of cancer stem cells, pushing them into proliferation and specifically sensitizing them to radiotherapy. In this context, targeting ATM seems very useful for killing cancer stem cells in the tumor. Recent studies have shown that CSCs may promote radioresistance by constitutive activation of a DDR started by the ATM protein. This idea was pioneered by Bao and collaborators in glioblastoma multiform (GBM) where cells expressing CD133 (Prominin-1), a marker for both neural stem cells and brain cancer stem cells, preferentially activate the DNA damage checkpoint in response to radiation. *In vitro* and *in vivo* experiments showed higher expression of activating phosphorylation of ATM, RAD17, CHK1 and CHK2 checkpoint proteins in these cells after IR treatment [127]. Indeed, CD133^+^ cells are characterized by preferential survival after irradiation, which may be overcome by treatment with CHK2 inhibitor. Consistent with these studies, two grade IV glioma cell lines displaying pronounced and poor stem phenotypes respectively, were pre-incubated with a non toxic concentration of the ATM inhibitors KU-55933 and KU-60019 and then irradiated, in order to improve the therapeutic efficacy of radiation on glioma stem cells (GSCs). GSCs were sensitized to radiation and radio-mimetic chemicals by ATM inhibitors, showing significantly reduced survival. No sensitization was observed after cell differentiation, indicating that ATM inhibitors specifically sensitize GSCs [128]. Very similar results have been obtained in breast cancer. In these studies, the subpopulation of CD44^+^/CD24^−/low^ cells, enriched for CSCs, of two breast cancer cell lines and the primary culture of patient breast cancer cells, demonstrated enhanced expression of phosphorylated ATM after radiation, which correlates with increased radioresistance. Using the ATM inhibitor KU-55933 they obtained significantly decreased radiation resistance of the CD44^+^/CD24^−/low^ subset isolated both from cell lines and from primary culture [129]. All together, these findings suggest a crucial role for ATM signalling in the regulation of survival of CSCs in response to DNA damage, suggesting that its inhibition may be exploited for the development of novel therapeutic strategies to extinguish CSCs.

Importantly, according to the wide range of functions exerted by ATM kinase activity, ATM inhibition may be useful to sensitize tumor cells also to other therapeutic approaches that do not target the DDR. In particular a positive role of ATM inhibition has been described in combined approaches with MET inhibitors [79] and ATM inhibition suppresses cell proliferation and induces apoptosis in cancer cells with overactivated AKT [78]. Conversely, ATM activation may promote cancer cell sensitivity to TRAIL [24]. TRAIL is an attractive therapeutic tool as it exerts a potent activity on cancer cells while it does not significantly affect normal cells [61,62]. As 60% of tumor cell lines and most primary cancer are resistant to TRAIL dependent apoptosis, several combined therapy approaches aimed to sensitize cells to TRAIL have been developed. One of the main features of TRAIL resistance is the aberrantly high level of c-FLIP proteins expression, as described in HCC and in other many tumors [61,62] and indeed their targeting enhances TRAIL sensitivity [66]. Evidence for the ability of several DNA damaging agents to dampen FLIP protein levels and sensitize cells to TRAIL have been provided by several laboratories [66]. We could show that ATM activity is required in HCC cells for c-FLIP proteins downregulation [27] suggesting the requirement of ATM activation to enable the DDR-dependent enhancement of TRAIL sensitivity at least in some contexts [130].

## 7. Concluding Remarks

Several pathways link ATM kinase expression and activity to the modulation of the balance between cell proliferation and cell death. Although several lines of evidence support a tumor suppressor function of ATM, in agreement with its canonical function as safeguard of genomic stability, the identification of ATM as a modulator of several additional cellular responses aberrantly activated in cancer has provided evidence for new and unexpected roles of ATM as a promoter of tumorigenic signals in particular contexts. This duality might be explained by the plethora of functions that ATM may exert, and could also be dependent on the specific genetic background. Future experiments will clarify the underlying molecular basis and more importantly will uncover its significance for cancer therapy.
